# Angle dependence as a unifying feature of root graviresponse modules

**DOI:** 10.1073/pnas.2506400122

**Published:** 2025-11-11

**Authors:** Suruchi Roychoudhry, Katelyn Sageman-Furnas, Harry J. Taylor, Iftekhar Showpnil, Chris Wolverton, Jiří Friml, Marta Del Bianco, Stefan Kepinski

**Affiliations:** ^a^Centre for Plant Sciences, School of Biology, University of Leeds, Leeds LS2 9JT, United Kingdom; ^b^Department of Biological Sciences, Ohio Wesleyan University, Delaware, OH 43015; ^c^Institute of Science and Technology Austria, Klosterneuberg 3400, Austria; ^d^Italian Space Agency, Rome 00133, Italy; ^e^Laboratory for Plant Biology, Food Production and Bioregenerative Systems, Centre for Space Life Sciences, Rome 00161, Italy

**Keywords:** gravitropism, root, angle dependence, auxin, PIN

## Abstract

Gravitropism, the patterning of postembryonic growth in relation to the gravity vector, allows plants to optimize the use of limited and nonhomogenous resources in their immediate environment. Since the current model of root gravitropism has not been able to integrate all aspects of the response (perception, response, and behavior), research on gravitropism has been dominated by different theories attempting to conceptualize each aspect individually. In this work, we sought to reevaluate all the main components of the root graviresponse through the lens of angle dependence. We show angle dependence in Cholodny–Went-based auxin asymmetry and growth response, which we tracked back to angle-dependent variation in PIN asymmetry and statolith sedimentation in the columella. Thanks to this approach, we were able to suggest distinct roles for PINs and columella cell tiers, and a potential function for auxin vertical flux through the columella. Our findings provide a unifying framework to further explore the mechanisms that regulate angle-dependent gravitropic response, with major implications of time-dependent features of root graviresponse.

## Significance

Gravitropism, the patterning of postembryonic growth in relation to the gravity vector, allows plants to optimize the use of limited and nonhomogenous resources in their immediate environment. In this work, we reevaluated all the main components of the root graviresponse through the lens of angle dependence. All the separate theories for root graviresponse are, therefore, likely conceptualization of the same mechanism and can be integrated in a cohesive model. Our findings provide a fundamental framework to further explore the mechanisms that regulate angle-dependent gravitropic response.

Plants have evolved the ability to adapt their postembryonic growth to the nonhomogenous distribution of resources in their environment through developmental responses called tropisms. Gravitropism is the ability of the plant to use the gravity field as a developmental cue to guide plant architecture (1). Root architecture is particularly important for the efficient uptake of water and nutrients below ground. The manipulation of root architecture to improve water and nutrient uptake and, enhance, carbon sequestration, is becoming an attractive strategy to tackle the modern challenges associated with a changing climate (2, 3). Crucial to the establishment of root architecture is nonvertical growth of lateral roots, which is tightly regulated by developmental pathways and external stimuli (4, 5). The capability to vary the growth angle of lateral roots requires the ability of these roots to discern different angles and elicit an angle-dependent response.

As described by the Starch-statolith theory (6), gravity is sensed thanks to dense amyloplasts (statoliths) in the specialized statocyte cells, which in the root are in the columella. When a root is displaced from its growth angle, statolith sedimentation to the new lower face of the cell (7, 8) triggers a signal transduction cascade that leads to the re-localization of auxin efflux proteins of the PIN-FORMED (PIN) protein family (9). In line with the Cholodny–Went theory, PIN polarization to the new lower side of the statocyte shifts the lateral efflux of auxin toward the lower side of the organ, where auxin inhibits cell expansion, causing bending (10, 11).

Kinematic studies of gravitropism have been an intensive field of study for more than a century (12). Initially, the Sine Law suggested that the magnitude of the gravitropic response was proportional to the component of the gravity vector that is perpendicular to the main axis of the organ (13). However, the Sine Law is unable to reproduce the behavior of plant roots gravistimulated at angles exceeding 90°. Indeed, freely responding roots normally display maximum bend rates between 120 to 130° and not 90° as a Sine Law behavior would imply, which led to the proposal of the Modified Sine Law (14). Despite these caveats, early studies demonstrated that there is angle dependence in the plant graviresponse. Evidence from the starchless mutant *pgm1* has suggested that, in roots, the angle-dependent gravitropic behavior and the formation of an auxin asymmetry (15, 16) rely on the presence of sedimenting statoliths. However, all aspects of the graviresponse have never been subjected to an integrated study to decipher their angle-dependent features.

Given the contrasting and incomplete evidence available in the literature, we sought to reevaluate angle dependence in the main components of the root graviresponse. Using tools for highly sensitive, quantitative reporting of auxin gradients, we found that gravitropism in *Arabidopsis* is angle-dependent and governed by quantifiable auxin gradients from stimulation angles as low as 30°, and that PIN3 and PIN7 play nonredundant additive roles in angle-dependent gravitropism. Taken together, our work provides a mechanistic framework for the formulation of a unifying theory of root gravitropism.

## Results

### Root Angle-Dependent Gravitropic Response across Angles.

**Fig. 1.**
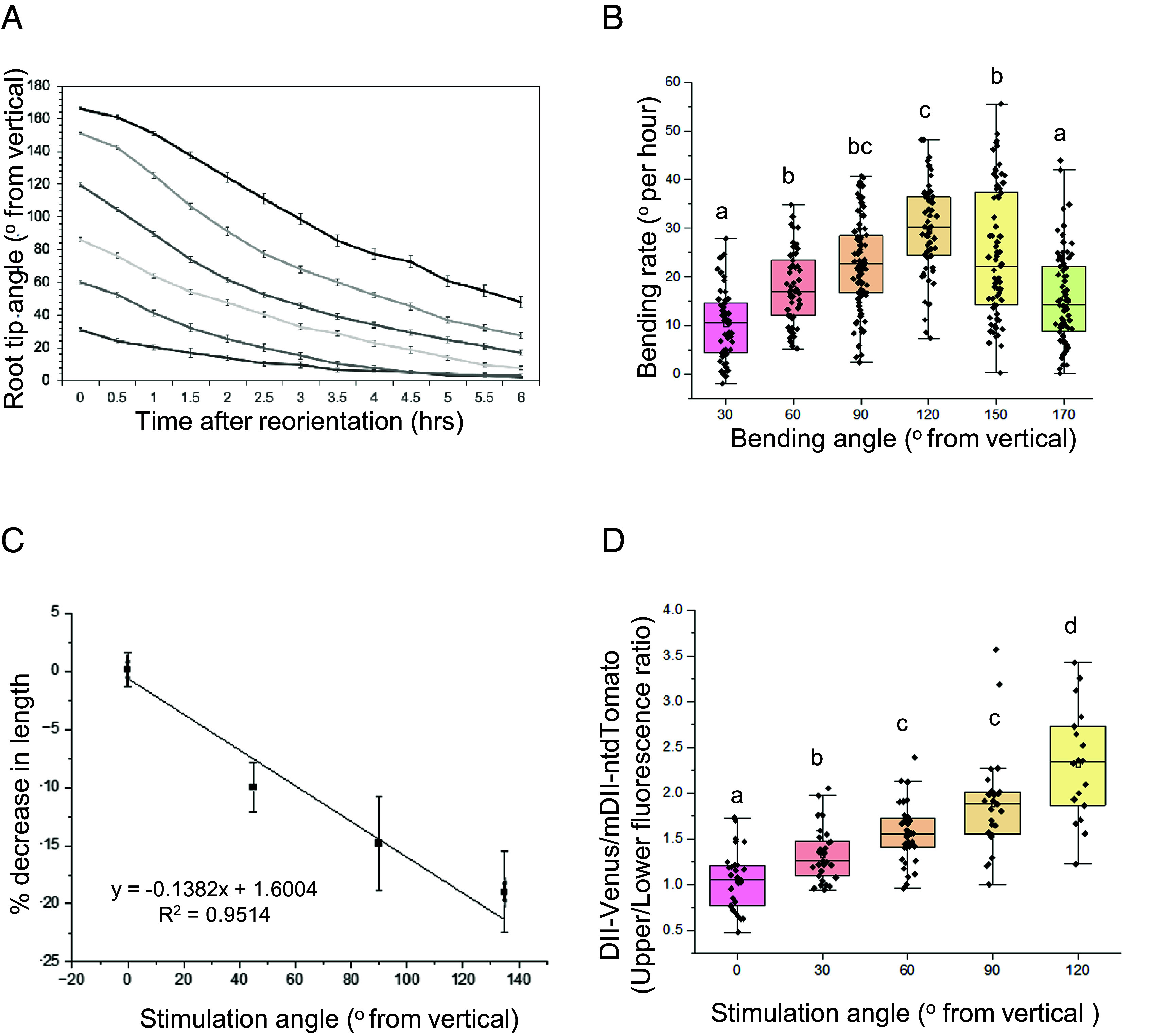
The root gravitropic responses in *Arabidopsis* is angle-dependent. (*A*) Gravitropic response kinetics assays of *Arabidopsis* at different stimulation angles (30° to 170°), bars represent SEM. (*B*) The first hour bending rate for each angle, n > 76 at each angle Letters represent significant differences of *P* < 0.01, one-way ANOVA with Tukey’s HSD test between all angle pairs. Median and quartile values are provided by the central line and box boundaries. The whiskers show 1.5× the interquartile range from the *Upper* and *Lower* quartiles. (*C*) Curvature of the root expressed as % decrease in length of the lower side compared to the upper side, n > 10 for each angle, bars represent SD. (*D*) Inferred auxin asymmetries of gravistimulated roots based on auxin measurements in only the same type of cells, n > 12 for each cell type and at each angle, letters represent *P* < 0.01, one-way ANOVA with Tukey’s HSD between all angle pairs when compared within the same cell type. Median and quartile values are provided by the central line and box boundaries. The whiskers show 1.5× the interquartile range from the *Upper* and *Lower* quartiles.

To assess the root gravitropic behavior, *Arabidopsis* seedlings were reoriented at different angles (30°, 60°, 90°, 120°, 150°, and 170°; n > 76) and imaged every 30 min, for 6 h (Fig. 1*A*). The magnitude of the gravitropic response was then expressed as average bend rate in the first hour after reorientation (Fig. 1*B*). The bend rate gradually increased between 30° and 120° reorientation angles and then decreased between 150° and 170°, with ~120° eliciting maximal response. These data are consistent with the observation that the angle-dependent behavior of freely responding roots does not follow a standard Sine Law (17–20).

For this reason, we focused on investigating the dynamics of root gravitropic behavior over the transition from above the horizontal (>90°) to the vertical (0°).

To confirm that the increase in differential growth rate was driven by a biophysical growth response, we quantified the percent reduction in the length of the lower side of the root as a parameter for curvature (Fig. 1*C*). This analysis revealed that the decrease in the length of the lower side significantly increased with reorientation angle, confirming that angle dependence is a sign of differential magnitude in the response and not, for example, in a greater persistence of the response though time. Since auxin has been shown to inhibit primary root growth in a dose-dependent manner (21), these data suggest that the angle-dependent gravitropic response may be due to differential accumulation of auxin.

### Angle Dependence Emerges from Auxin Asymmetry and a Lack of Redundancy between PINs.

To study the relationship between stimulation angle, bending response, and auxin gradients, we used the ratiometric *R2D2* auxin reporter, which, combined with vertical imaging, allows for sensitive quantitative inference of auxin levels (22). In this reporter, auxin accumulation is manifested as the reduction of yellow signal relative to the red signal. Due to the inherent differences in *R2D2* signal between epidermal trichoblast and atrichoblast (*SI Appendix*, Fig. S1 *A* and *B*), we compared fluorescence ratios between the same cell type (*SI Appendix*, Fig. S1*C*). Using this method, we found that quantifiable auxin gradients were present at stimulation angles as low as 30°, and that auxin gradients were correlated with stimulation angles between 30° and 120° (Fig. 1*D*). Taken together, these data support the idea that angle-dependent auxin gradients dictate the magnitude of root gravitropic response.

**Fig. 2.**
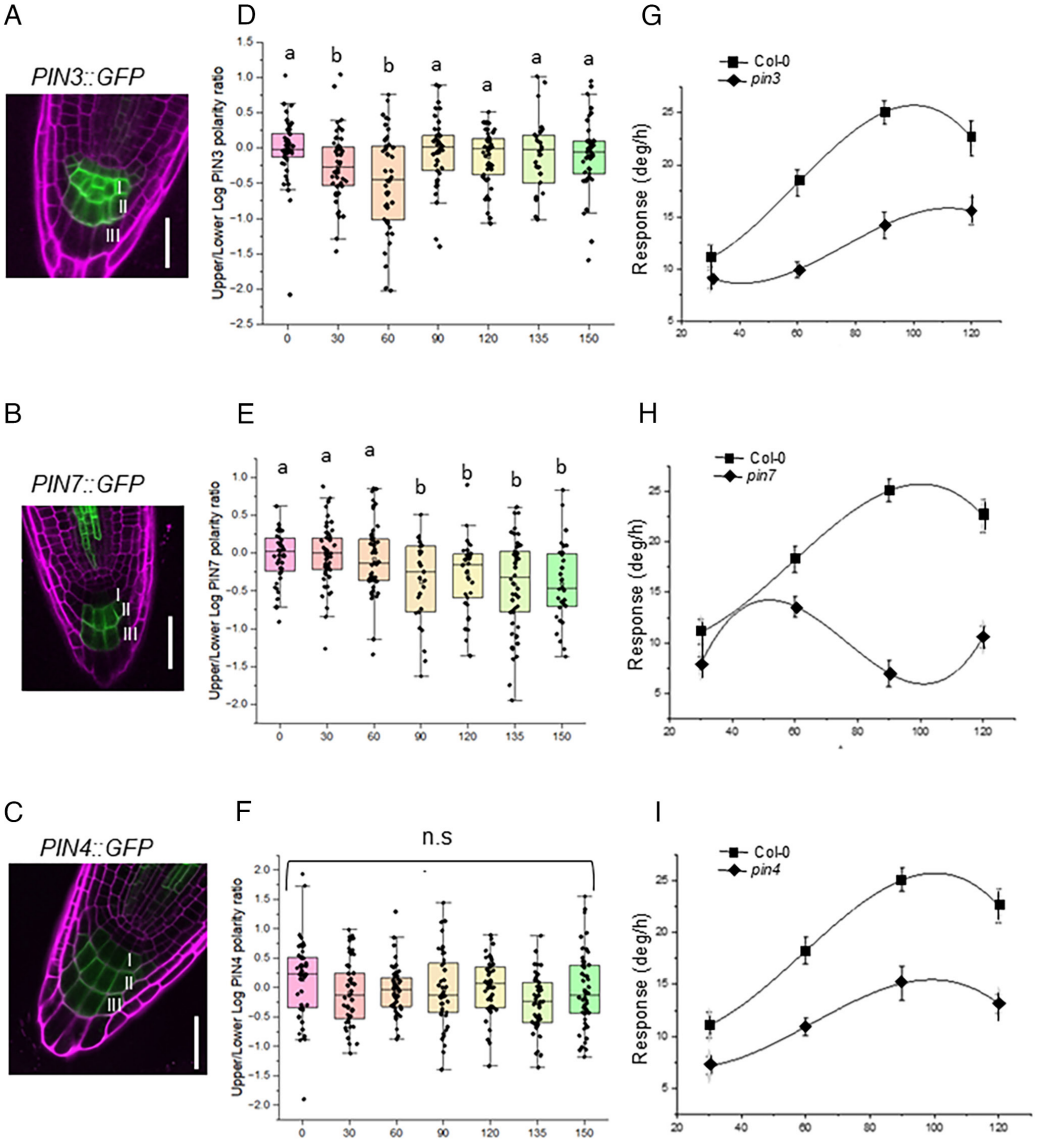
Nonoverlapping functions of PIN3, PIN7, and PIN4 in root gravitropism. (*A-C*) PIN3::GFP, PIN7::GFP, PIN4::GFP expression in columella cells. PIN3 is expressed strongly in the upper two tiers (I and II) of columella cells, PIN7 is expressed at higher levels in the lower two tiers (II and III) of columella cells, while PIN4 is expressed in all three columella tiers. (Scale bar, 30 μM.) (*D–F*) Quantification of PIN3::PIN3:GFP, PIN7::PIN7:GFP, PIN4::PIN4:GFP membrane fluorescence within upper and lower plasma membranes of primary root columella cells in *Arabidopsis* seedlings gravistimulated at a range of angles. 15 to 20 roots were gravistimulated at each angle with 8 to 12 cells analyzed per root. Median and quartile values are provided by the central line and box boundaries. The whiskers show 1.5× the interquartile range from the *Upper* and *Lower* quartiles. Different letters indicate *P* values < 0.01 as calculated through one-way ANOVA followed by posthoc Tukey’s HSD test (*G–I*) Graviresponse kinetics of wt (Col-0), and the pin3 (*G*), pin7 (*H*), and pin4 (*I*) loss-of-function mutants under constant gravistimulation stimulus using the ROTATO system. 15 to 20 roots were gravistimulated at each angle. Bars represent SEM.

To clarify the role of columella cells in determining the angle-dependent auxin asymmetry, we first performed detailed kinematic studies of PIN polarization in *Arabidopsis* primary roots. *PIN3* and *PIN7* are expressed in the columella (10, 23) and are essential for root graviresponse. Using vertical-stage confocal microscopy, we analyzed the PIN expression pattern and polarization in the columella cell membranes in the *pPIN3::PIN3:GFP* and *pPIN7::PIN7:GFP* translational lines (Fig. 2). In our experimental conditions, we found that *PIN3* was expressed strongly in the upper columella cell tiers, while *PIN7* was expressed strongly in the lower two columella cell tiers (Fig. 2 *A* and *B*). After reorientation at different angles, both PIN3 and PIN7 polarized in the direction of gravity 30 min after reorientation in an angle-dependent manner (Fig. 2 *D* and *E*). Interestingly, while PIN3 showed prominent polarization at lower angles (Fig. 2*D*), PIN7 showed asymmetric distribution only at angles above 60° (Fig. 2*E*). These results are consistent with previously published data for PIN3, but not PIN7 polarization at 45° (4, 24), although this discrepancy is likely due to difference in growth conditions and bioimaging protocol. Both PINs did not show any angle-dependent internalization and/or polarization away from the distal columella cell membranes (*SI Appendix*, Fig. S3). Overall, these data suggest a potential differential contribution of PIN3 and PIN7 to gravitropic response at different stimulation angles.

Despite their central role in mediating the gravitropic response, plants carrying single *PIN* loss-of-function mutations display only mild phenotypes (24). This has been suggested to be caused by the functional redundancy between members of the PIN family. Using a constant gravitropic stimulation system (18), we were able to closely assess the root gravitropic response in *pin3* and *pin7* single mutants. This analysis revealed that the *pin3* mutant displays an overall flattening of the typical bell-shaped response, with a decrease in the magnitude of bend rate over all the angles tested (Fig. 2*G*). On the other hand, *pin7* showed a normal phenotype up to 60°, with a severely impaired gravitropic response at higher angles (Fig. 2*H*). Analysis of the *pin3pin7* double mutant under constant stimulation revealed a pattern of response similar to that of *pin3* single mutant, but more severe (*SI Appendix*, Fig. S2). This suggests that while PIN3 might play a central function in mediating an angle-dependent response, PIN7 still plays a redundant as well as nonredundant, additive role in root graviresponse. In this context, it is also important to note that previous studies have shown that loss-of-function mutations in single and multiple PIN proteins lead to ectopic upregulation of other PIN proteins (25, 26).

Among the PINs expressed in the root, *PIN4* is detected in the stem cell niche, basally in provascular and epidermal cells, and in the first tier of the columella (27) (Fig. 2*C*). We assessed the phenotype of the *pin4* loss-of-function mutant under constant gravitropic stimulation and found it to be similar to *pin3* (Fig. 2*I*). However, the analysis of the p*PIN4::PIN4:GFP* translational marker line after gravistimulation at different angles revealed that PIN4 failed to polarize significantly at any angles (Fig. 2*F*). Quantification of *PIN4:GFP* membrane fluorescence within distal plasma membranes of gravistimulated roots also showed no significant variations at different stimulation angles (*SI Appendix*, Fig. S3). These data indicate that the *pin4* mutant phenotype may not be due to an abnormal response downstream of statolith sedimentation.

### Statolith Sedimentation Determines Angle-Dependent PIN Polarization.

It has been previously shown that the *Arabidopsis* starchless mutant *pgm1* retains a basal gravitropic response and no angle dependence15. To confirm the role of statoliths in the angle-dependent polarization of PIN proteins, the *pPIN3::PIN3:GFP, pPIN4::PIN4:GFP,* and *pPIN7::PIN7:GFP* marker lines were introduced in the *pgm1* mutant background. Vertical-stage confocal microscopic analyses revealed that PIN3, PIN4, and PIN7 polarization was abolished in a *pgm1* mutant background (*SI Appendix*, Fig. S4 *A*–*C*). This confirms that angle dependence is specifically mediated by statolith sedimentation and downstream PIN relocalization.

**Fig. 3.**
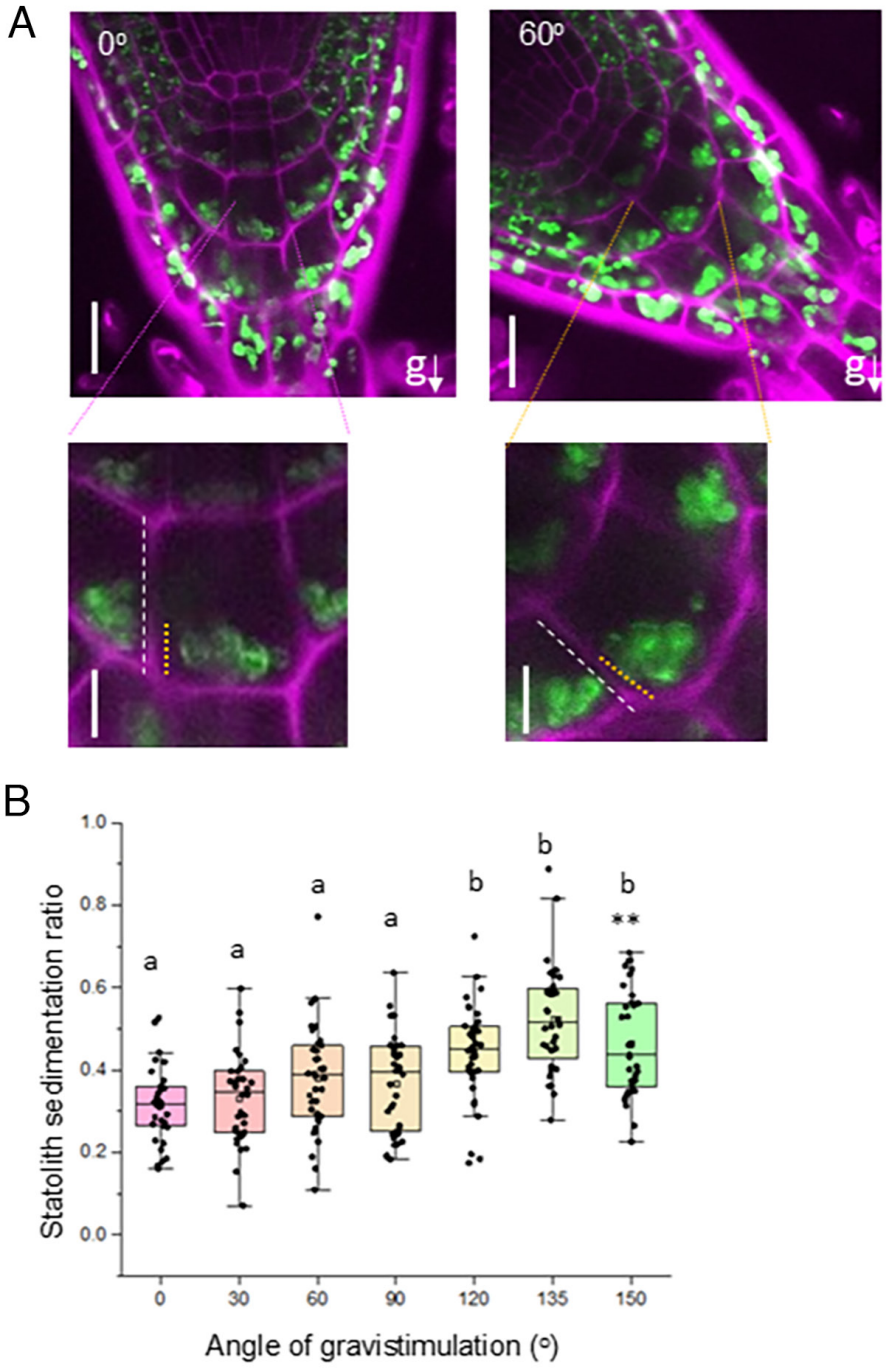
Statolith sedimentation ratio is proportional to the angle of gravistimulation. (*A*) Statolith position in 5-d-old vertically growing (*Left* and *Inset*) primary roots of *Arabidopsis* expressing the YFP tagged plastid marker Pt-YK. Reorientation by defined angles results in positioning of the statoliths toward the new physical bottom of the cell (*Right*). Statolith sedimentation ratio was defined as the length of the cell wall in contact with the statoliths (yellow dashed line) divided by the total length of the cell way (white lines). Scale bar, 20 μM in *Upper* panels and 10 μM in the *Lower* panels (*B*) Statolith sedimentation ratio is proportional to the angle of gravistimulation and peaks at 135o. 15 to 20 roots were gravistimulated at each angle with 4 to 6 cells analyzed per root. Median and quartile values are provided by the central line and box boundaries. The whiskers show 1.5× the interquartile range from the *Upper* and *Lower* quartiles. Letters indicate *P* values < 0.01, from one-way ANOVA followed by posthoc Tukey’s HSD test.

Previous studies have suggested a proportionality between stimulation angles and overall statolith sedimentation (28, 29). To better assess statolith sedimentation in our system, we reoriented *Arabidopsis* roots expressing the fluorescent plastid marker Pt-YK (30) at different angles. In columella cells imaged 5 min post reorientation, in accordance with previous studies, statoliths tended to sediment in aggregates (31). Statolith sedimentation ratio along the columella cell membrane was estimated as the length of the cell membrane in contact with statoliths, divided by the total length of the columella cell (Fig. 3*A*). Quantification of statolith sedimentation in all the columella cell tiers demonstrated that sedimentation ratios increased in an angle-dependent manner up to 135° but declined at 150°, as the almost vertically inverted orientation led to increased sedimentation on the upper, horizontal cell wall (Fig. 3*B*), consistent with a maximum bend rate at around 135°. In the *pgm1* mutant, Pt-YK labeled statoliths showed an abnormal, filamentous morphology (*SI Appendix*, Fig. S5*A*). Consistent with previous studies (32), statolith sedimentation did not occur (in an angle-dependent manner, or otherwise) in the starchless *pgm1* mutant background even 15 min after gravistimulation (*SI Appendix*, Fig. S5*B*).

To further assess statolith sedimentation, we quantified, statolith sedimentation ratios along the distal columella cell membrane and found that these decreased in an angle-dependent manner (*SI Appendix*, Fig. S6*A*). Further, we also quantified the velocities of sedimenting statoliths using time-lapse live cell imaging. Roots expressing YFP or CFP tagged plastid fluorescent markers [Pt-YK or Pt-CK (30)] were reoriented at different angles and imaged from 60 s up to 5 min postreorientation, when statoliths would settle on the new basal surface of the cell. Statolith sedimentation velocity increased generally in an angle-dependent manner up to 120°, before declining again at 150° (*SI Appendix*, Fig. S6*B*). These data could justify the initial observation of ~120° eliciting maximal response and opens the possibility of a complex interaction between angle-dependent magnitude and time factors within the columella.

## Discussion

Gravitropism has been conceptualized through three main theories: the starch-statolith theory for sensing, the Cholodny–Went model for signal transduction and growth control, and the law of angle dependence for behavior. Previous work on the starchless mutant *pgm1* had already suggested that the angle-dependent gravitropic behavior of plant roots (15) and the formation of an auxin asymmetry (16) rely on the presence of sedimenting statoliths. Here, we demonstrate that angle dependence in Cholodny–Went-based auxin asymmetry and growth response can be traced back to angle-dependent variation in PIN protein asymmetry in the gravity-sensing columella cells and statolith sedimentation, even at low stimulation angles. Our results agree with previous work that showed that angles as small as 15° were sufficient to trigger a gravitropic bending in *Arabidopsis* roots (18). By showing that all major components of the gravitropic response display angle-dependent behavior, we provide a mechanistic framework toward the formulation of a unifying theory of root gravitropism.

In this work, we were able to uncover features of the root graviresponse using the more sensitive R2D2 marker and taking into account the variation in signal among epidermal cell types. Importantly, we attempted to assess angle dependence independently of time. Previous work using the *Arabidopsis* DII-Venus reporter had proposed a “tipping-point” model of root gravitropism (16), where roots would reach the vertical thanks to the persistence of the auxin response, while the gradient is lost half-way through the graviresponse. The results here obtained by addressing other reorientation angles would suggest that the tipping-point model might actually be highlighting time-dependent features of the graviresponse. The existence of time-dependent features would imply that the gravitropic behavior of roots reoriented at a lower angle is different from roots that reach that same angle after being reoriented at a higher one. Constant gravitation stimulus is the only available experimental approach that can circumvent the change in stimulation angle as a root responds to gravity. Interestingly, constant stimulus studies have shown a constant response at different angles but a different angle dependence behavior, more similar to the Sine Law, compared to freely responding roots (18). This indicates that a maximum bend rate above 90° could be due to time-dependent features, e.g., response speed. For example, here we show that statolith sedimentation velocity is consistent with a maximum bend-rate above 90°. Moreover, it was shown that plastid sedimentation rates are heterogeneous within the columella (33). Additional layers of complexity to the resulting root gravitropic kinetics could arise from other elements of the response, like cell expansion rate and a potential proprioceptive response (34). More work will therefore be needed to understand the contribution of possible time-dependent features of the graviresponse to the overall kinetic (35).

The analysis of the PIN polarization and mutant combinations under constant stimulation presented here has revealed nonredundant, additive roles in root graviresponse for PIN3, PIN7, and PIN4. PIN3 polarization is most prominent at lower angles, however its phenotype suggests that its function is required for the generation of an asymmetric auxin gradient across a wide range of angles. The discrepancy between free response and constant stimulus could indicate a possible time-dependent feature of PIN3 polarization. PIN7 polarization is instead more prominent at angles exceeding 60°, in accordance with its mutant phenotype under constant gravitational stimulus. Laser ablation studies have previously revealed that columella cell tiers play different roles with respect to gravity sensing (presentation time) versus graviresponse (final tropic growth response of the root). The removal of tiers I and II had the greatest effect, while ablation of tier III slows the kinetics of bending but has no effect on sensitivity suggesting that the signal was produced in tier I and II, and amplified in tier III (33). Conversely, it could be concluded, from the further evidence produced in this work, that PIN3 polarization could mediate the bulk of the response from tier I and II, while PIN7 would amplify the signal at angles >90° from tier II and III. Unfortunately, the lack of R2D2 expression in the lateral root cap and the delay in DR5 response upon auxin treatment (36) hinder our ability of directly prove this hypothesis. Moreover, PIN expression is influenced by auxin variations in a tissue-specific manner, and the loss of even single PINs can lead to the ectopic overexpression of other PINs (25, 26). Mathematical modeling, and more refined auxin markers and genetic approaches will therefore be needed to verify the distinct role of PINs in the root graviresponse, circumventing gene redundancy and complex regulatory pathways.

Auxin distribution within the root meristem and columella is likely important for a normal gravitropic response. PIN4 mediates the formation of an auxin sink around the quiescent center and the input of auxin from the vasculature to the columella (27). The loss-of-function mutation of *PIN4* causes a flattening of the auxin gradient, with a decrease in auxin response in the columella. While *pin4* has a gravitropic phenotype very similar to *pin3*, our analysis showed a lack of gravi-dependent repolarization of PIN4:GFP in both lateral and basal membranes. This suggests that the *pin4* mutant phenotype could be the symptom of an overall disturbed auxin distribution, which could affect *PIN3* and *PIN7* expression and/or the creation of upper/lower auxin gradient. Moreover, it has been suggested that PIN proteins, including PIN4, PIN3, and PIN7, form protein complex containing homo- or heterodimers (9). PIN4 could therefore act as a general regulator of cellular auxin efflux in a gravity-independent way. Interestingly, though the ablation of whole tiers abolishes curvature, laser ablation of vertical files of central columella cells does not elicit the same effect (33). This could suggest that the vertical flux within the columella, albeit indirectly, is important for graviresponse. While the levels of PIN4, PIN3, and PIN7 localized to the distal plasma membrane do not vary in response to gravistimulation (*SI Appendix*, Fig. S3), an impaired auxin flux to lower tiers could contribute to the phenotype of the loss-of-function mutants. In the future, an approach based on live imaging and computational modeling will be required to study the complex effects of the overall fluxes and distribution of auxin within the root meristem during the root graviresponse.

Our statolith sedimentation analyses ties into the most recent hypothesis in plant gravitropism: the “position-sensor hypothesis” (20, 37). This hypothesis, first developed in the context of shoot gravitropism, states that statocytes act as clinometers, wherein the position of the statoliths in relation to the plasma membrane induces corresponding auxin fluxes. Here, we show that PIN polarization is proportional to the degree of statolith sedimentation in the root gravisensing cells. This link is supported by recent studies demonstrating the role of LAZY proteins (38, 39). It has been suggested that LAZYs translocate to the plasma membrane thanks to statolith sedimentation, where they seem to act as positional sensors for PIN polarization and/or activation, presumably through RLD1 (40) and D6PK-dependent mechanisms (41). Thus, taken together, our data provide molecular support for the position-sensor hypothesis in the root.

**Fig. 4.**
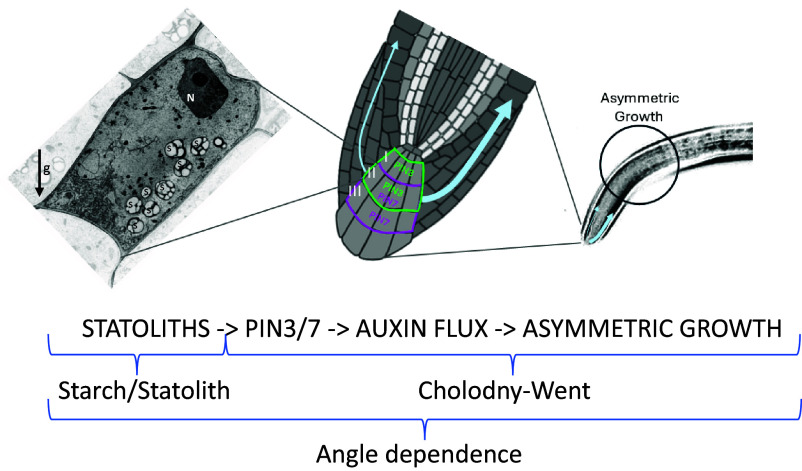
Angle dependence as a unifying theory of root gravitropism. In the root, gravity is sensed thanks to dense amyloplasts (statoliths) in specialized columella cells (*Left*) (Starch-statolith theory). Upon disturbance from the vertical, statolith sedimentation to the new lower face of the cell triggers a signal transduction cascade that leads to the relocalization of auxin efflux proteins of the PIN protein family, asymmetric auxin flux and cell expansion, and bending (Cholodny–Went theory) (*Central* and *Left*). According to our unifying framework, all the main components of the root graviresponse are linked by angle dependence. Thanks to our approach, we were also able to show that different PINs (PIN3 and PIN7) and columella cell tiers (I, II, II) play distinct roles in establishing the asymmetric auxin gradient at different angles and postulate the importance of the vertical flux through the columella. *Left* panel is graphic representation of an ideal columella cell as it would be if imaged with a transmission electron microscope, adapted from Perbal and Driss-Ecole, 2013. g = Earth’s gravity vector; s = statoliths; N = nucleus.

The work presented here shows that all the main components of the root graviresponse, from statolith sedimentation to response, are linked by angle dependence. All the separate theories for root graviresponse (starch/statolith and Cholodny–Went) are, therefore, likely conceptualization of the same mechanism and can be integrated thanks to a cohesive angle dependence framework (Fig. 4). This conceptualization allowed us to postulate distinct roles of PINs and columella cell tiers and the existence of time-dependent features in the root graviresponse. Overall, these observations represent an important step forward in our understanding of the biology of gravitropism and toward the exploration of major outstanding questions in the field.

## Methods

### Plant Material and Growth Conditions.

Seeds of *Arabidopsis thaliana* in a Columbia (Col-0) background were used in this study. The following lines have been previously described: *pin3*, *pin4*, *pin7*, *pin3pin7* (10, 27), *pPIN3::PIN3:GFP* (24), *pPIN4::PIN4:GFP* (25), *pPIN7::PIN7:GFP* (24), PT-YK, PT-CK (30), and *pgm1* (42).

Seeds were surface sterilized using chlorine gas4. Following sterilization, seeds were sown in chambered slides containing solidified *A. thaliana* salts (ATS) growth medium. A 1 cm well was cut into the top of each chambered slide using a sterile scalpel, and 2 to 3 seeds were transferred in the corner between gel and glass slide. Seeds were kept at 4 °C for at least 2 d and transferred to environmental growth cabinets, with each slide positioned in a vertical orientation to maintain primary roots parallel with the gravity vector. Seeds were incubated in standard tissue culture conditions under 20 ± 2 °C, long day (16 h light/8 h dark cycle), and 400 to 500 μmol m^−2^ s^−2^ light conditions, for 5 to 7 d.

For constant gravitropic stimulus experiments, plants were grown as previously described18. Briefly, seeds were surface sterilized in ethanol solution (70% v/v), followed by washes in 95% ethanol. Seeds were sown on 60 mm petri dishes containing solid half-strength MS media, refrigerated for 1 to 5 d before being placed under continuous illumination with cool-white fluorescent lights (80 μmol m^−2^ s^−1^) for 4 to 5 d at 22 ± 2 °C.

### Reorientation Assays and Confocal Microscopy.

Vertically grown 5-d-old Col-0 seedlings were gravistimulated in infrared light and imaged at 30-min intervals using a converted infrared camera with an 830 nm filter (43) for the analysis of gravitropic response kinetics. For curvature analysis, 4 h after reorientation, whole roots were cut with the agar and carefully mounted on glass slides with 1.5 mM propidium iodide and imaged on an inverted Zeiss LSM 880 Axio Imager 2 confocal. Following 1 h of acclimation, 5-d-old *R2D2* seedlings were imaged through the root mid-plane using a vertical-stage confocal microscope setup (*SI Appendix*, Fig. S1*A*). Roots were then gravistimulated at different angles (30° to 120°) and imaged after 40 min. Relative auxin levels were calculated for each nucleus of the upper and lower root epidermis as in Roychoudhry et al.’s work (2023) (4). Briefly, excluding the lateral root cap, nuclear fluorescence was measured in ten consecutive epidermal cells within the two outermost flanking cell files, beginning from the root tip for each root. Experiments were performed three times with at least ten root tips for each orientation per experiment. Nuclear fluorescence intensity was measured across both GFP and mTomato channels. For each nucleus, the ratio of GFP/mTomato signal was determined. Since we found inherent differences in GFP/mTomato signal between hair and nonhair cells (*SI Appendix*, Fig. S1*B*), we compared ratios between the same cell type (Fig. 1*D*).

Curvature was expressed as the delta, in %, between the length of the inner vs. outer root perimeter across the length of the gravitropic bent (*SI Appendix*, Fig. S7). Measurements were performed using ZEN Blue software (Zeiss) with a segmented line. Col-0 plants were gravistimulated at 0°, 45°, 90°, and 135° in the same set-up used for reorientation experiments. These angles were chosen as being representative of a smaller angle, of the horizontal, and of an angle slightly above that inducing the fastest bend rate.

For PIN protein polarization and statolith sedimentation experiments, roots were stained with propidium iodide and gently placed into an LSM800 inverted confocal microscope (Zeiss) rotary stage at 0° GSA, using a 40× (numerical aperture 1.2) ultrasound immersion objective. For *PIN:GFP* lines, a Z-series of columella cells was acquired for each root by capturing 25 to 55 slices at 1 µm intervals, using a 488 nm laser at 4× averaging. Roots were initially fixed onto a backboard at angles ranging from 0° to 150° using a two-axis spirit level, then left for 30 min to allow PIN polarization to take place. The angle of reorientation was then maintained as slides were positioned in the LSM800 confocal for image acquisition. The same images were used to quantify statolith sedimentation ratios as well as *PIN:GFP* fluorescence from the distal membrane at different angles of gravistimulation. For distal membrane data, the statolith sedimentation ratio was quantified as the ratio of the distal membrane in contact with statoliths divided by the total length of the distal membrane. Distal *PIN:GFP* fluorescence was quantified as the ratio between the fluorescence at the distal membrane divided by the total fluorescence across all membranes for each columella cell. For quantification of statolith sedimentation velocity using PT-YK and PT-CK plastid marker lines, seedlings were illuminated with a 500 ± 10 nm or 405 ± 10 nm laser, respectively, at angles ranging from 0° to 150° for 50 frames (5.8 to 13.4 s/frame depending on microscope performance). Scaling per pixel (0.185 μm × 0.185 μm) and resolution (512 × 512 pixel) were consistent between statolith experiments.

To image the expression domain of PIN3/4/7::GFP, seedlings were grown on vertically oriented ATS medium plates for 5 d and then mounted in propidium iodide solution on glass slides and standard cover slips using an LSM880 inverted confocal microscope (Zeiss). Images were captured at 40× magnification using the 488 nm and 543 nm lasers for GFP and propidium iodide, respectively.

### Image Analysis.

To discern the apical–basal targeting of PIN3 and PIN7, measurements were performed using ImageJ software (NIH). The fluorescent intensity across internal membranes of columella cells was quantified using the approach by Roychoudhry et al., 2023 (4). Using the “Plot profile” function in ImageJ, the *x*-axis point of maximal intensity in the PI channel was identified as the cell wall. The GFP fluorescence was measured and calculated across each cell membrane on either side of the cell wall. The log signal ratio before and after reorientation was calculated as the average of signal intensity ratios per cell for each angle. Ten replicates of 3 to 6 seedlings were analyzed.

Statoliths were tracked using IMARIS (Bitplane Scientific Software) and ZEN Blue (Zeiss) software, with 5 to 10 centrally positioned statoliths per root tracked across C1-C4 tiers. Each track was set to a maximum gap size of 1.5× the diameter of an individually tracked statolith, and each track was manually checked so that track jumping within statolith aggregates did not overestimate an individual plastid movement through time. Where statoliths moved too far through the z-axis to be tracked, a minimum of 15 frames was used as the benchmark for calculating velocity. To compensate for any object drift and ensure that time-resolved velocity of individual statoliths was kept consistent between images, a cell membrane was tracked in the PI channel. The horizontal velocity (Vx) and vertical velocity (Vy) outputs provided the total statolith velocity using the trigonometric function.

where *t* is the length of time between each frame in the series. Direct motion of statoliths could then be calculated by subtracting the resultant vector (Δz) for the plasma membrane from the Δz for the statoliths. Statistical analysis was calculated using MS Excel 365 software (Microsoft). Statolith sedimentation speeds were calculated by measuring the length of a statolith aggregate in microns and waiting until they had spread an equivalent distance following reorientation on the (new) basal membrane, using a digital caliper.

### Constant Gravitropic Stimulus.

Constant gravitropic stimulus experiments were performed using the ROTATO image analysis and feedback system, as previously described18. Briefly, seedling roots were positioned on a rotatable stage in the center of the axis of rotation and custom software was used to maintain the tip segment in a fixed orientation relative to gravity through image analysis coupled to a stepper motor. As the root continued to undergo gravitropic curvature, its response kinetics were captured as the rotation required to constrain the tip at the prescribed angle.

### Statistical Analysis.

Unless stated above, all statistical analyses were performed in SPSS (IBM). The normality of data was assessed (Kolmogorov Smirnoff test with Lilliefors correction), and a one-way ANOVA was performed with a post hoc Tukey’s HSD test.

## Supplementary Material

Appendix 01 (PDF)

## Data Availability

All study data are included in the article and/or *SI Appendix*.
