# Supplementary material for: Angle dependence as a unifying feature of root graviresponse modules
Source: Proc Natl Acad Sci U S A. 2025 Nov 11;122(46):e2506400122. doi: 10.1073/pnas.2506400122 (PMC12646229; doi:10.1073/pnas.2506400122)
Supplement: Supplementary file 1 — Appendix 01 (PDF) [file pnas.2506400122.sapp.pdf]

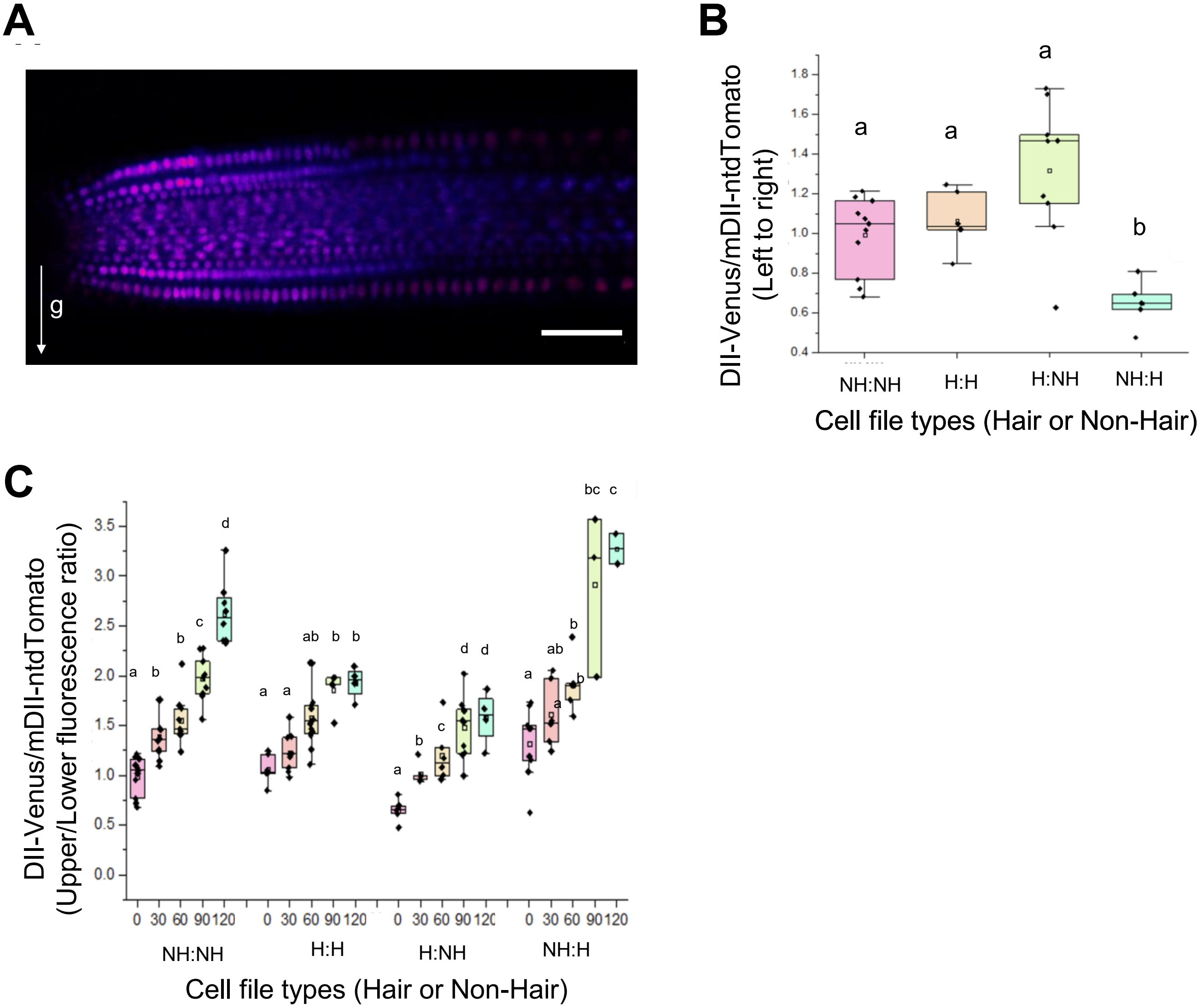

Figure S1: Angle-dependent auxin asymmetries in the Arabidopsis primary root tip can be quantified using *R2D2*. (A) An example image used for analysis of inferred auxin asymmetries using the *R2D2* reporter. Scale bar = 50 uM. (B) Venus and ntdTomato fluorescence of *R2D2* comparing auxin asymmetries between Hair and Non-Hair cell types in vertically growing roots, n > 6. Letters indicate p values <0.01, from one-way ANOVA followed by posthoc Tukey's HSD test. (C) Inferred auxin asymmetries of roots gravistimulated for 40 minutes at different angles. Letters indicate p values <0.01, from one-way ANOVA followed by posthoc Tukey's HSD test for comparisons between fluorescence ratios for different angles of stimulation for each cell file type analyses. Median and quartile values are provided by the central line and box boundaries. The whiskers show 1.5x the interquartile range from the upper and lower quartiles.

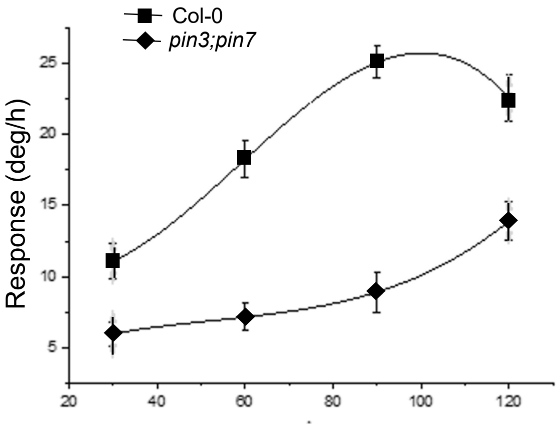

Figure S2: Graviresponse kinetics of the *pin3;pin7* loss-of-function mutant under constant gravistimulation stimulus using the ROTATO system. Col-0 was used as wild-type control. 15-20 roots were gravistimulated at each angle. Bars represent s.e.m.

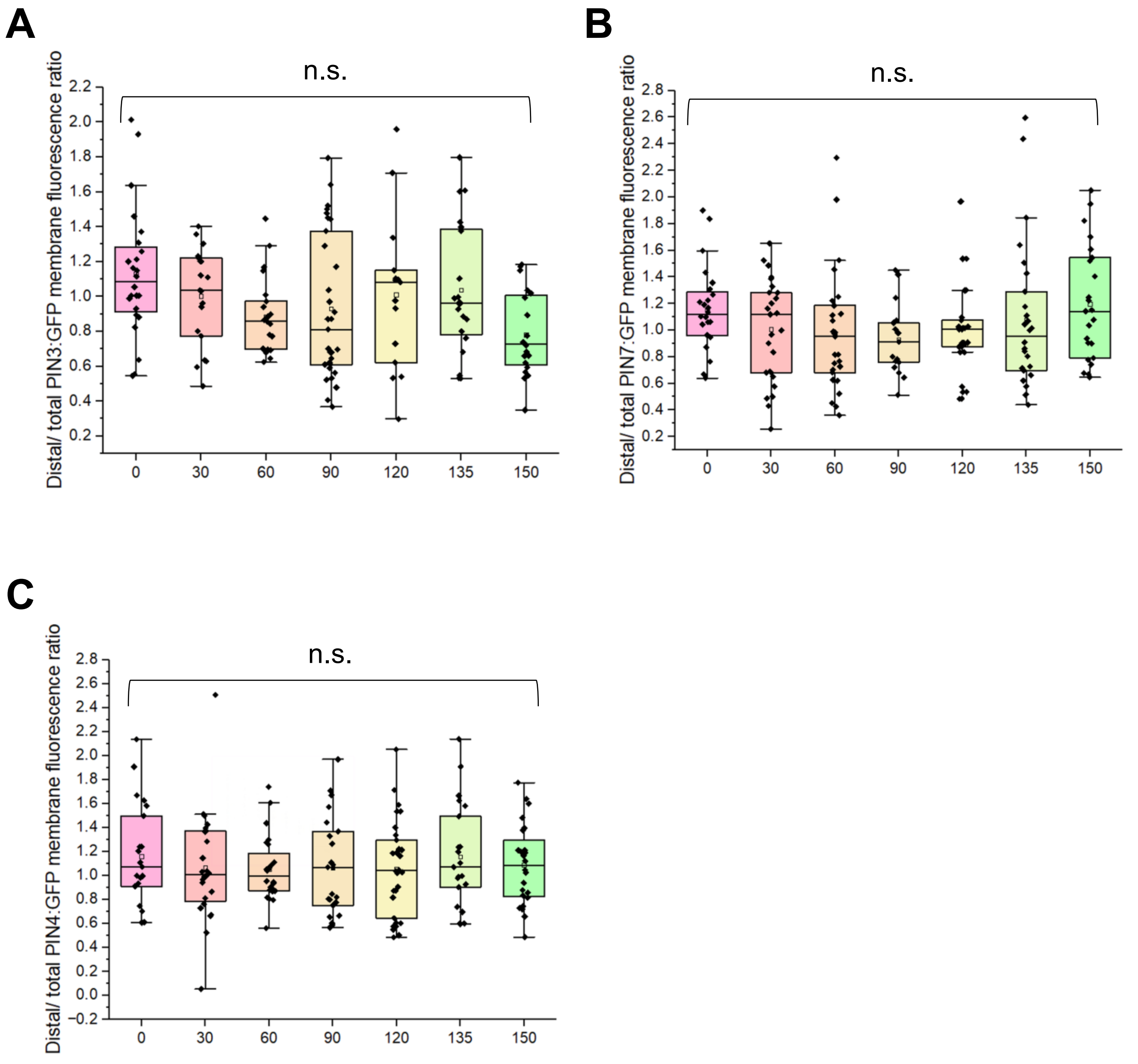

Figure S3: Analysis of PIN3/4/7::GFP levels in the distal membrane at different angles of gravistimulation. Quantification of PIN3::PIN3:GFP, PIN7::PIN7:GFP, PIN4::PIN4:GFP membrane fluorescence within distal plasma membrane of primary root columella cells in Arabidopsis seedlings gravistimulated at a range of angles. No significant variations of PIN3/4/7:GFP (A-C) was found to occur at different angles of gravistimulation in the distal membrane of root columella cells. Membrane levels were normalised by the PIN3/4/7:GFP total cellular content. Significance was calculated by one-way ANOVA. 15-20 roots were gravistimulated at each angle with 8-12 cells analysed per root. Median and quartile values are provided by the central line and box boundaries. The whiskers show 1.5x the interquartile range from the upper and lower quartiles. 'n.s'. indicates 'not significant'.

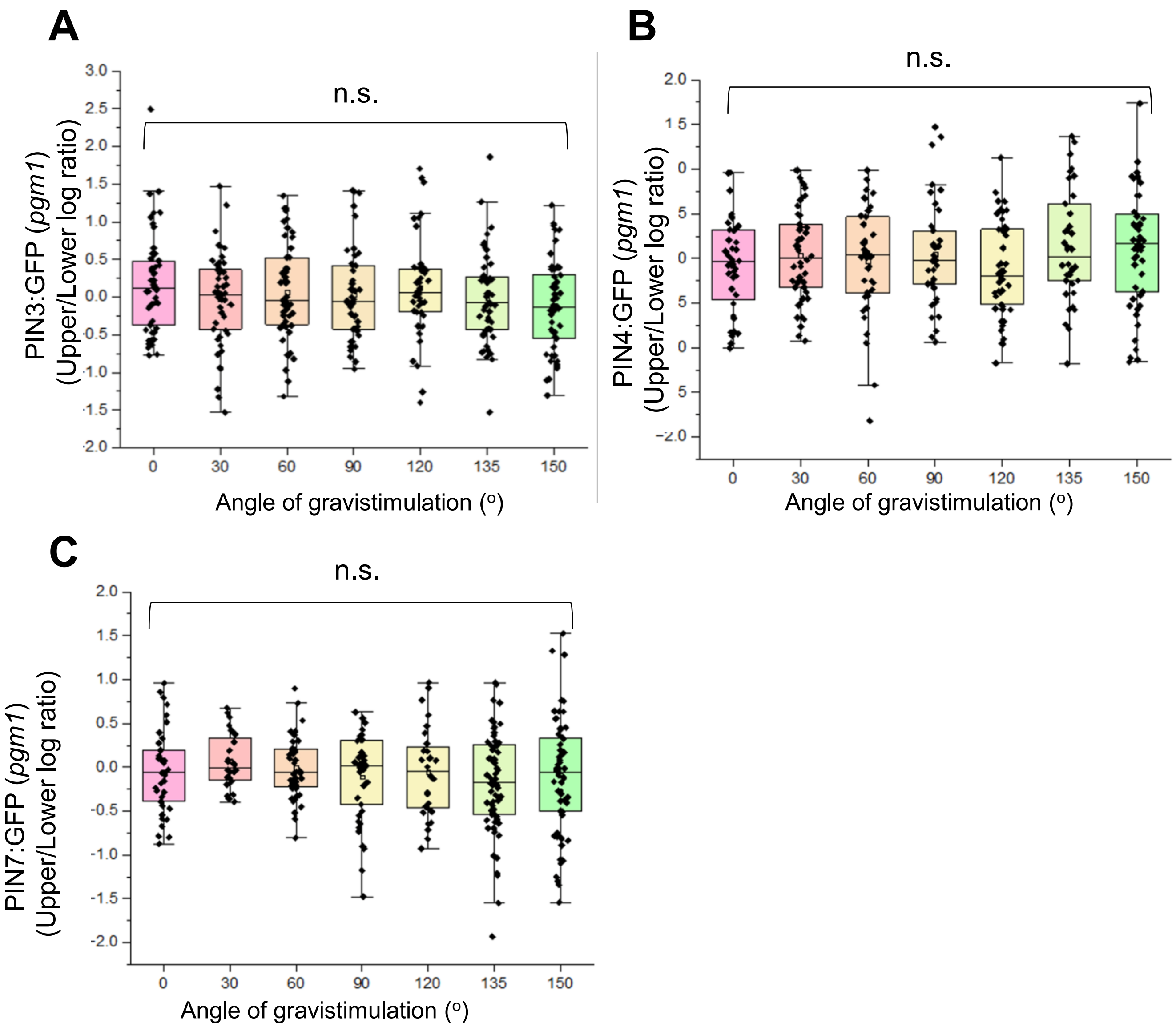

Figure S4: Analysis of PIN3/4/7 polarization in the *pgm1* mutant background. The angle-dependent polarization of PIN3/4/7:GFP (A-C) was found to be absent in the *pgm1* mutant background. No significant differences (indicated by n.s) were found in the polarisation ratios of PIN3/4/7::GFP at different angles of gravistimulation in the *pgm1* mutant background as calculated by one-way ANOVA. 15-20 roots were gravistimulated at each angle with 8-12 cells analysed per root. Median and quartile values are provided by the central line and box boundaries. The whiskers show 1.5x the interquartile range from the upper and lower quartiles.

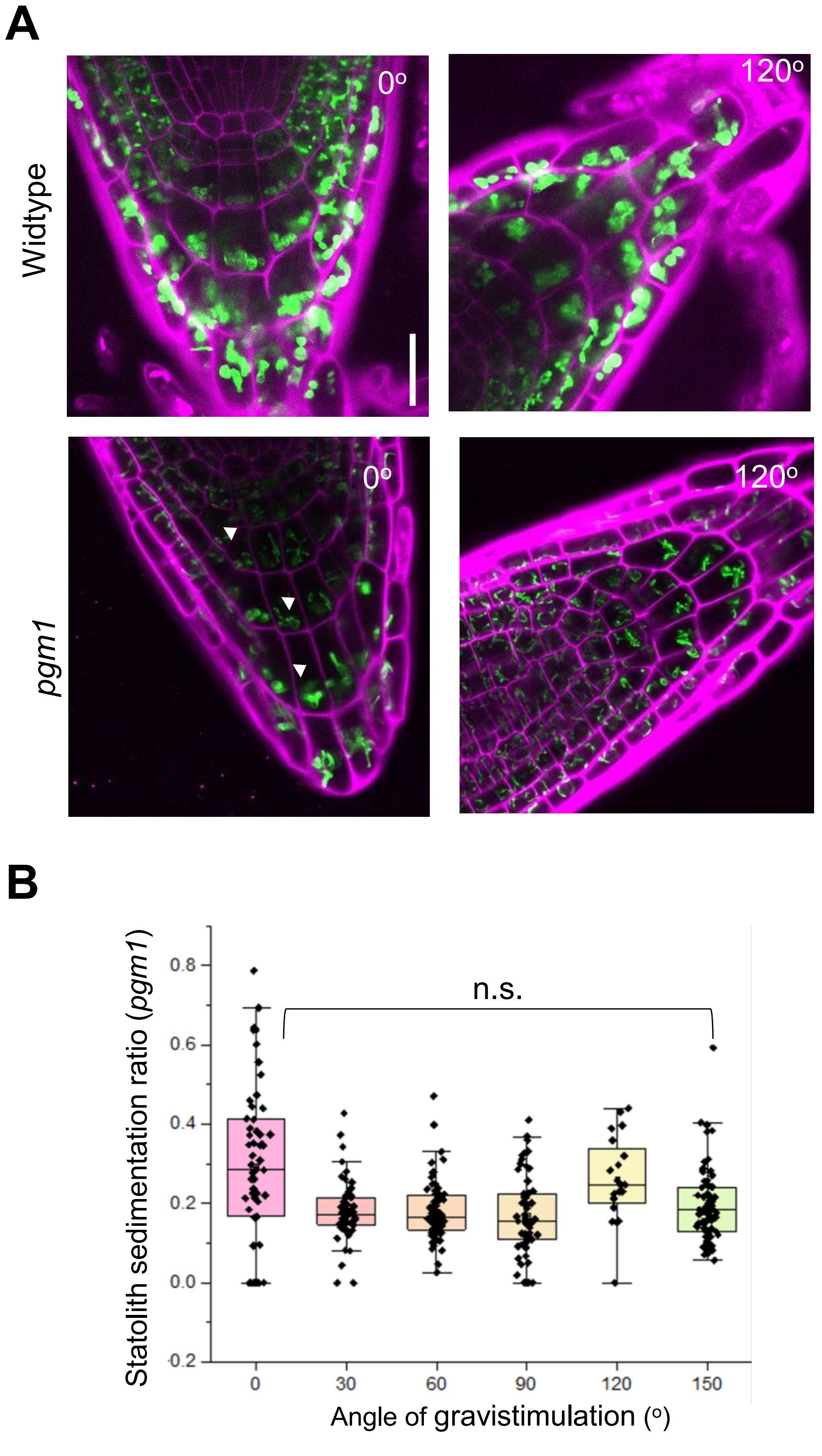

Figure S5: Statolith sedimentation and morphology are impaired in the *pgm1* mutant. (A) Representative images of plastid morphology and sedimentation after gravistimulation at defined angles in WT and *pgm1* mutant plants expressing the Pt-YK YFP plastid marker. Statoliths were smaller and filamentous in the *pgm1* mutant (white arrowheads), and statolith sedimentation did not occur in columella cells in the *pgm1* mutant following gravistimulation. Scale bar = 20 uM. (B) Statolith sedimentation is completely abolished in the *pgm1* mutant background indicated by no significant differences in statolith sedimentation ratios across different angles of stimulation as calculated by one-way ANOVA. 15-20 roots were gravistimulated at each angle with 4-6 cells analysed per root. Median and quartile values are provided by the central line and box boundaries. The whiskers show 1.5x the interquartile range from the upper and lower quartiles.

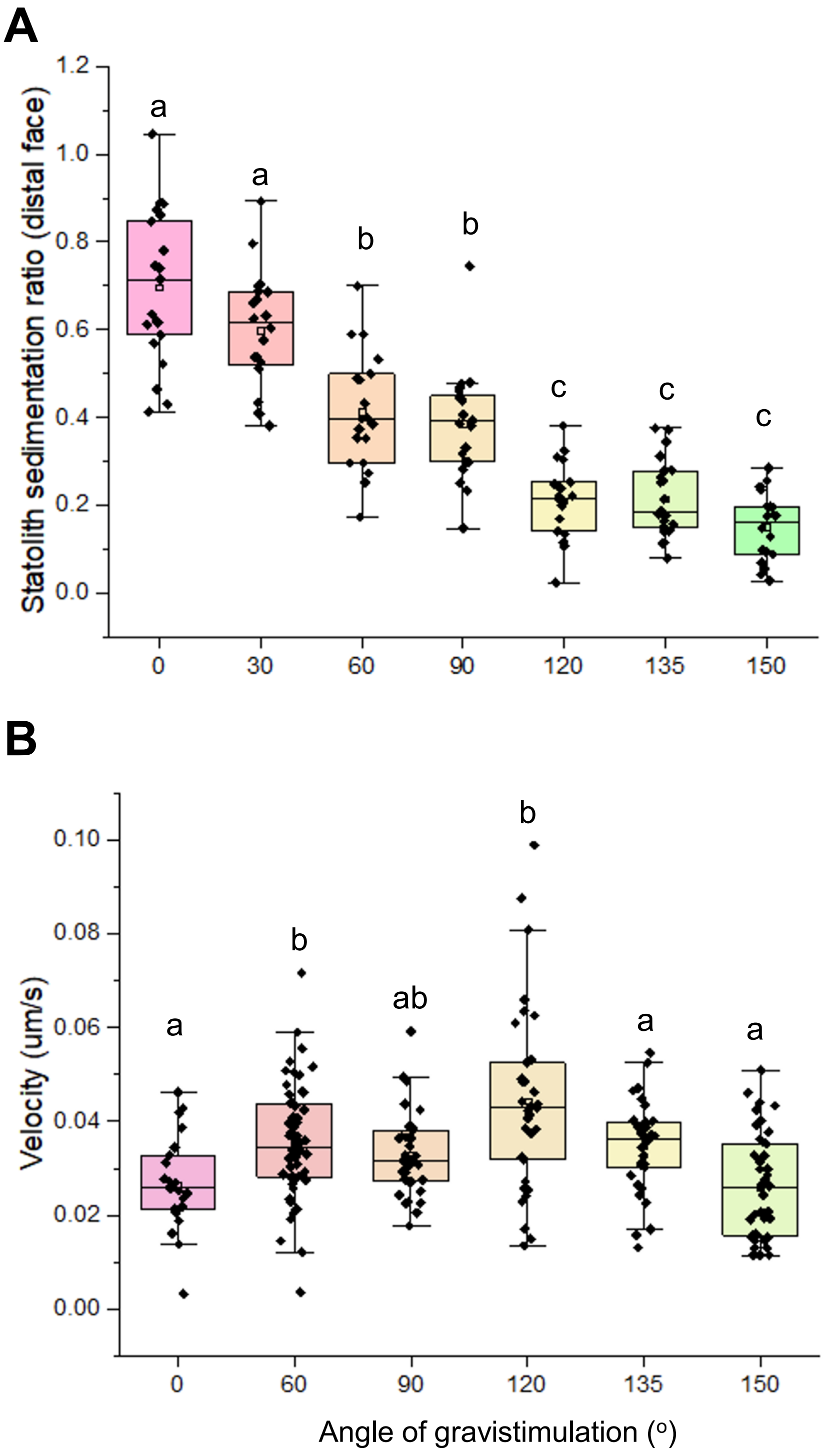

Figure S6: Analysis of statolith sedimentation. (A) Statolith sedimentation ratio of the distal membrane at various angles of gravistimulation show angle-dependent behaviour. 15-20 roots were gravistimulated at each angle with 8-12 cells analysed per root. (B) Statolith sedimentation velocity was quantified in columella cells of 5-day-old Arabidopsis roots expressing the plastid marker Pt-YK. Roots were gravistimulated by the defined angle on the rotating stage of a vertically mounted confocal microscope, and images of statoliths were captured every 5s for 15 mins. 10-15 statoliths were tracked at each angle. The experiment was repeated three times. Letters indicate p values <0.01, from one-way ANOVA followed by posthoc Tukey's HSD test. Median and quartile values are provided by the central line and box boundaries. The whiskers show 1.5x the interquartile range from the upper and lower quartiles.

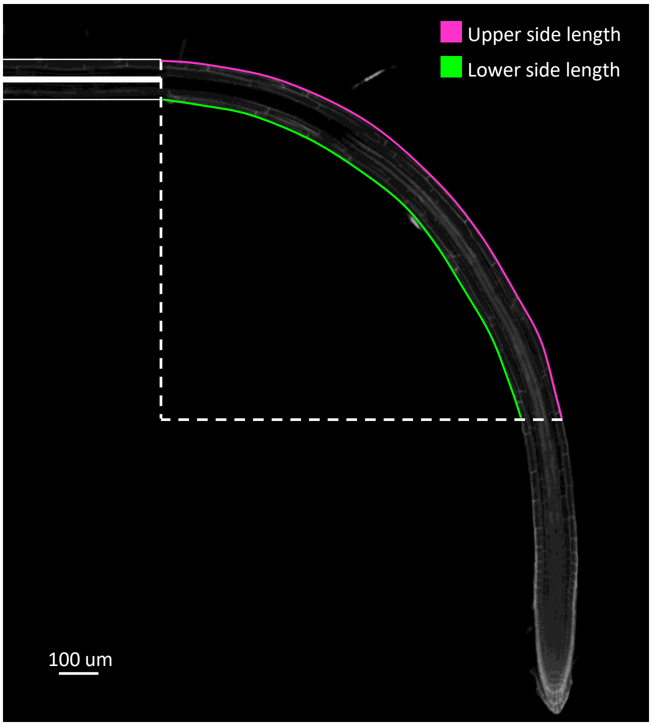

Figure S7: Visual representation of upper and lower side measurement.
(A) Plants were grown on vertical ATS-agar plates and gravistimulated in the dark for 2 h. Col-0 plants were gravistimulated at 0°, 45°, 90°, and 135° in the same set-up used for reorientation experiments. These angles were chosen as being representative of a smaller angle, of the horizontal, and of an angle slightly above that inducing the fastest bend rate. A root reoriented at 90° is shown as example. Plants were then carefully mounted on microscope glass slides with 1.5 mM propidium iodide and imaged on an inverted Zeiss LSM 880 Axio Imager 2 confocal. Composite, z-stack images were captured to cover the tip, the whole curvature, and part of the straight root pre-curvature. The ZEN Blue software (Zeiss) was used to stitch the images and measure the length of the upper (magenta) and lower (green) outer sides of the epidermal cells with a segmented line. The start of the curve is identified by the point where the outer edges of the root stop overlapping with the prosecution of the straight pre-curvature axis. The end of the curvature is identified by either the last hair primordium within the curvature or by the point where the outer edges of the root stop overlapping with the prosecution of post-curvature root axis.

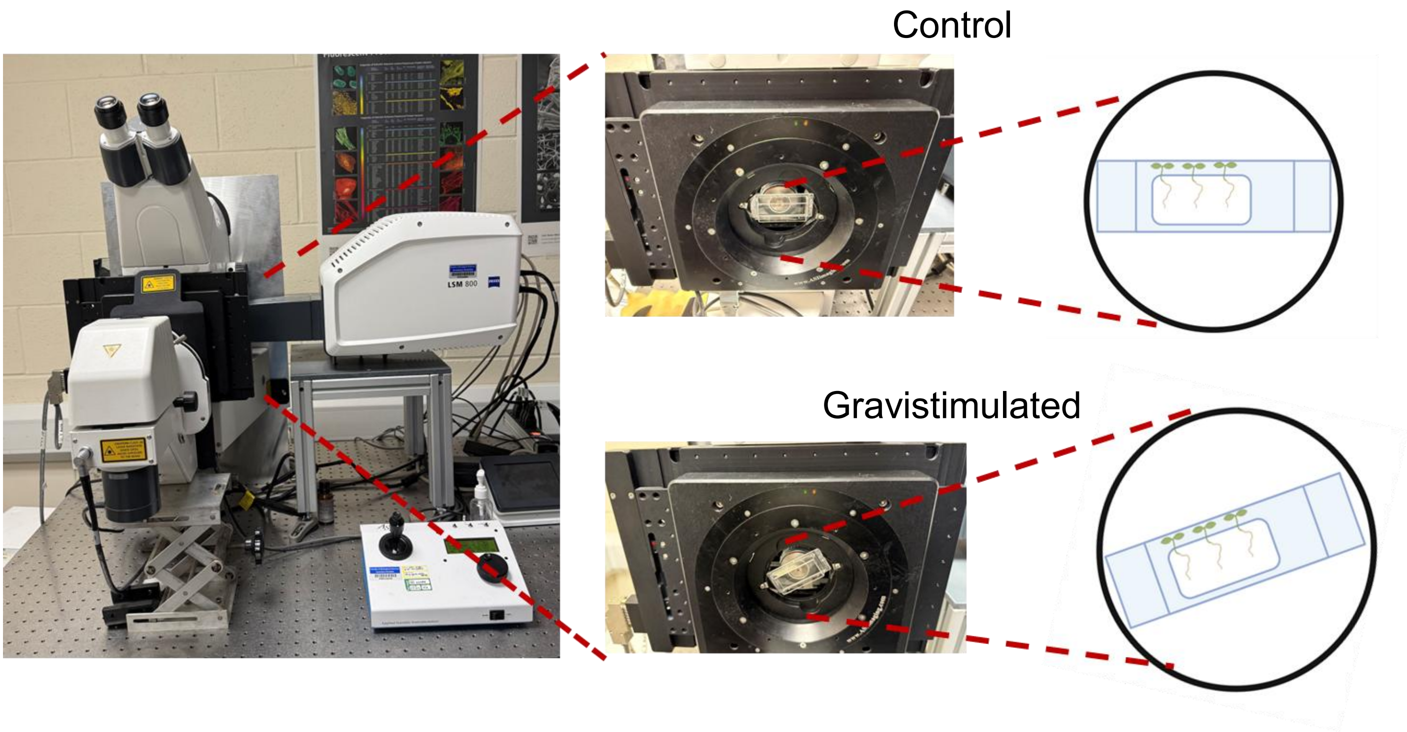

Figure S8: Graviresponse in PIN loss-of-function double mutants. Schematic of the imaging setup used to capture confocal images of control and gravistimulated roots containing reporter lines and fluorescent markers. Briefly, roots were grown on a chambered slide overlayed with a thin layer of ATS medium and imaged in vertical orientations or at multiple angles of gravistimulation using a rotational stage on a vertically mounted LSM800 confocal microscope (Zeiss).

Figure	Description	Test	F statistic	Df	P value	n
1A	Bending rate	One way ANOVA	31.476	5	1.11E-16	47-53
1D	DII Venus	One way ANOVA	33.625	4	1.11E-16	19-24
2C	PIN3 GFP	One way ANOVA	4.453	6	3.00E-04	37-55
2F	PIN7 GFP	One way ANOVA	5.264	6	3.62E-05	33-55
2I	PIN4 GFP	One way ANOVA	2.504	6	2.02E-02	43-57
3B	Statolith angle	One way ANOVA	24.383	6	1.11E-16	28-36
S1B	Hair non hair total	One way ANOVA	8.806	3	3.00E-04	7- 10
S1C	NH:NH all deg	One way ANOVA	60.929	4	1.11E-16	7 -12
S1C	H:H all degrees	One way ANOVA	16.302	4	1.51E-07	5 - 8
S1C	NH:H all degrees	One way ANOVA	12.307	4	1.15E-05	5 - 9
S1C	H:NH all degrees	One way ANOVA	16.435	4	3.05E-06	3 - 7
S3A	PIN3 GFP distal membrane	One way ANOVA	2.303	6	3.75E-02	18-24
S3B	PIN7 GFP distal membrane	One way ANOVA	1.384	6	2.24E-01	23-25
S3C	PIN4 GFP distal membrane	One way ANOVA	0.283	6	9.44E-01	22-27
S4A	PIN3 GFP x pgm1	One way ANOVA	0.875	6	5.14E-01	41-47
S4B	PIN4 GFP x pgm1	One way ANOVA	2.295	6	3.49E-02	37-44
S4C	PIN7 GFP x pgm1	One way ANOVA	0.268	6	4.35E-01	39-46
S5B	Statolith angle x pgm1	One way ANOVA	3.368	6	1.46E-02	55-72
S6A	Statolith distal membrane	One way ANOVA	58.491	6	1.11E-16	22-33
S6B	Statolith velocity	One way ANOVA	12.431	5	1.65E-08	26-41

Table S1: Statistical parameters for data analysis
